# Mandibular swelling as the initial presentation for renal cell carcinoma: A case report

**DOI:** 10.1016/j.ijscr.2020.04.061

**Published:** 2020-05-11

**Authors:** Roy Zhang, Chang Woo Lee, Shadi Basyuni, Vijay Santhanam

**Affiliations:** a University of Cambridge School of Clinical Medicine, Box 111 Cambridge Biomedical Campus, Hills Road, Cambridge, CB2 0SP, Cambridge, United Kingdom; b Department of Oral and Maxillo-Facial Surgery, Cambridge University Hospitals, Hills Road, Cambridge, CB2 0QQ, United Kingdom

**Keywords:** Renal cell carcinoma (RCC), Metastasis, Mandibular swelling, Case report

## Abstract

**Introduction:**

Renal cell carcinoma (RCC) is the most common malignant tumour of the kidney. It usually presents in an occult manner, rarely with the classical triad of haematuria, abdominal mass and abdominal pain. Up to a third of patients have metastasis on presentation and only a few case reports have involved the mandible.

**Presentation of case:**

We present the case of a renal cell carcinoma that presented, in a 56-year-old lady, with mandibular swelling as its main clinical manifestation. This patient presented with a 3-month history of right sided facial swelling, associated with pain and intermittent paraesthesia to the right side of the tongue and lower lip. Imaging of the mandible revealed a lesion that had caused complete destruction of the right condyle, coronoid and ramus. Ultrasound guided biopsy revealed the nature of the mass to be metastatic renal cell carcinoma. Subsequent computed tomography (CT) imaging of the abdomen and pelvis confirmed the presence of a tumour in the right kidney. Due to the advanced nature of the disease, radical treatment was not suitable, and the patient passed away 11 months after diagnosis with palliative care.

**Discussion and conclusion:**

Whilst mandibular swelling is usually benign, it should be kept in mind that orofacial symptoms can be the initial presentation of systemic disease. Persistent swellings with infection ruled out, or those causing cranial nerve palsy, should be investigated further.

## Highlights

• Chronic unilateral parotid swellings require further investigation.
• Malignancies should be excluded in any facial swellings causing cranial nerve palsies.
• Orofacial symptoms can be the initial presentation of systemic disease.
• Exclude malignancy in gross haematuria presenting in adults.

## 1 Introduction

Renal cell carcinoma (RCC), arising from renal tubular epithelium, is the most common malignant tumour of the kidney [1]. It classically presents as the triad of haematuria, flank pain and a palpable abdominal mass, but in reality, this only occurs in a minority of cases. Other common presenting features include non-specific symptoms such as fatigue, weight loss, loss of appetite and anaemia. Management is predominantly surgical and early diagnosis is paramount to success [2]. Newer targeted therapies have been shown to be beneficial.

Up to a third of patients have metastasis on presentation with common sites being regional lymph nodes, lungs, liver, bones and the brain. Metastasis to the orofacial region has been well documented in the skin, subcutaneous tissue, parotids and paranasal sinuses. Involvement of the mandible is extremely rare with only a few reports over the last 25 years [[3], [4], [5], [6]].

Our report is the first where mandibular involvement was the primary presenting feature. The presented case report adheres to the SCARE guidelines [7].

## 2 Presentation of case

A 56-year-old female was referred to hospital by her dentist with a 3-month history of right sided facial swelling. This had been slowly increasing in size and was associated with intermittent paraesthesia to the lower lip and tongue. Only mild pain was reported, satisfactorily relieved by paracetamol and ibuprofen when needed. Her general medical practitioner (GP) had previously prescribed a course of antibiotics that had not improved the facial swelling.

Her medical history was remarkable only for hypertension and anxiety, for which she was taking lisinopril and citalopram respectively. She was on no other regular medication and had no known allergies. She was a current smoker on presentation. She denied any weight loss or reduction in appetite. Her past medical and family history were otherwise insignificant.

**Fig. 1:**
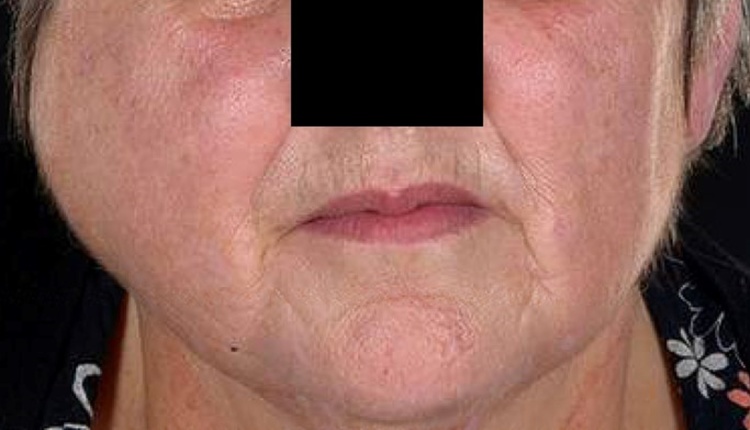
Right-sided mandibular swelling.

Examination showed a marked right-sided swelling (Fig. 1). The lesion was tender to palpate and, despite it being firm and fixed, there were no overlying skin changes. The swelling extended 4 cm laterally and was associated with a reduced mouth opening of 28 mm (inter-incisal). Intraorally, it was also tender to palpate the external oblique ridge and adjacent teeth. Facial and trigeminal nerves appeared intact.

### 2.1 Diagnostic assessment

**Fig. 2:**
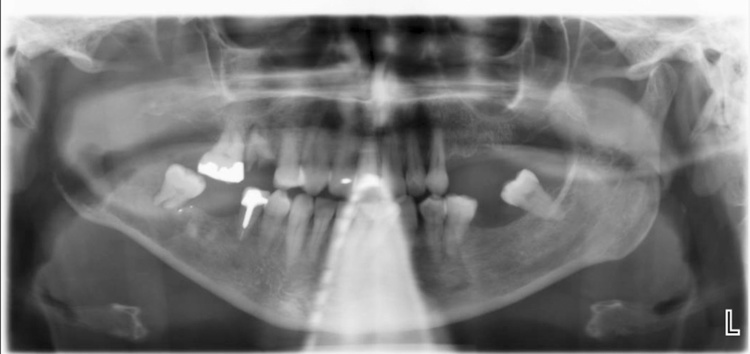
Orthopantomogram (OPG) demonstrating destruction of the right condyle, coronoid and ramus.

**Fig. 3:**
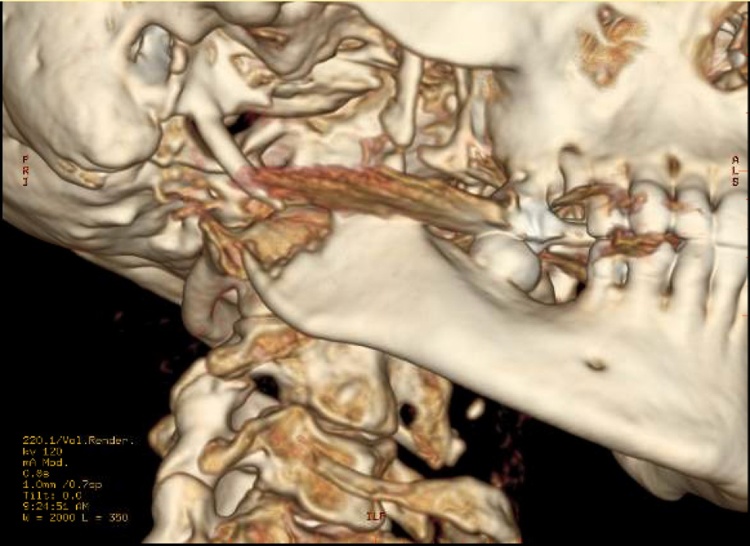
CT scan demonstrating a large enhancing soft tissue mass with central necrosis, measuring 57 × 53 mm.

An orthopantomogram (OPG) was performed (Fig. 2). This plain film radiograph showed complete destruction of the right condyle, coronoid and ramus with an irregular non-corticated extension of the lesion into the body of the mandible. This area was further investigated with CT (Fig. 3). This showed a large enhancing soft tissue mass with a central area of necrosis measuring 57 × 53 mm. The lesion displaced the parotid laterally and extended to the pterygoid muscles.

**Fig. 4:**
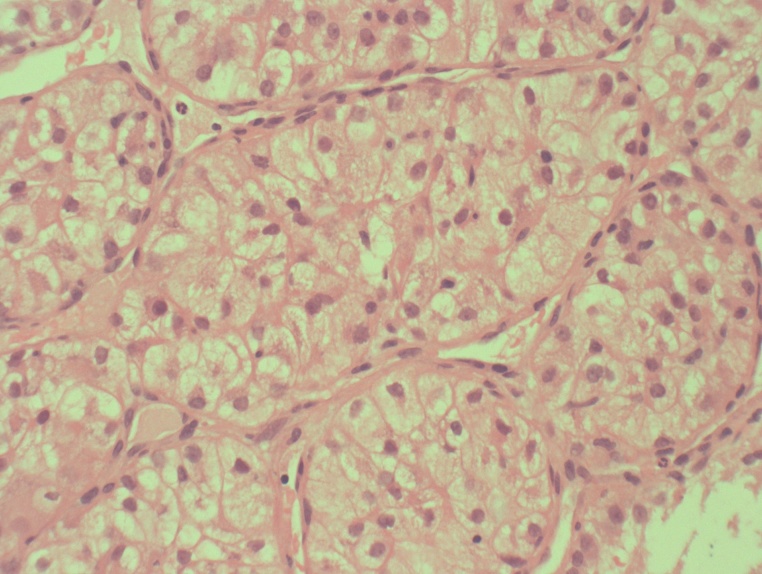
Histopathology revealed nests of cells with abundant cytoplasm that stained positive on immunohistochemistry for CD10, PAX8 and renal cell markers.

The area was sampled via needle biopsies at the initial appointment. Histopathological investigation revealed nests of cells with abundant cytoplasm that stained positive on immunohistochemistry for CD10, PAX8 and renal cell markers (Fig. 4).

A subsequent CT abdomen/pelvis was arranged, which identified the primary as a right sided renal tumour of middle and lower pole (14.7 × 13.2 cm) with no extension into right renal vein or IVC.

### 2.2 Differentials

The initial presentation of facial swelling (Fig. 1) has multiple differentials. Parotitis could cause a swelling in the same region and this is what her GP was initially treating her for. Similar facial presentations can also be due to primary tumours involving the parotid, the mandible (sarcomas or odontogenic tumours) and the oral mucosa. The persistence of her facial swelling, despite antimicrobial therapy, suggested a non-infective cause that necessitated further investigation.

Causes of radiolucencies in the mandible (Fig. 2) are also extensive. Similar radiographic appearance is seen in ameloblastomas, keratocystic odontogenic tumours or malignancies such as sarcomas. The extensive bony destruction and history of neuropathy in this case was suggestive of a more sinister aetiology. Irrespective of clinical judgement, a biopsy is needed to investigate radiolucencies of this size and nature.

Following immunohistochemical staining, it became evident that this lesion was a metastatic renal cell carcinoma. This was confirmed when the primary was identified on CT imaging of the right kidney.

### 2.3 Therapeutic intervention

Following discussions at both the Head & Neck and Urology multidisciplinary team meetings, it was ultimately decided that the patient was to be managed with palliative intention. On further questioning, the patient reported that she had one episode of gross haematuria 7 months prior to the development of her facial swelling. This resolved with a course of antibiotics.

Her main problem was the discomfort from the enlarging mandibular mass. It was decided that a 6-week cycle of sunitinib (50 mg daily) should be commenced without delay. If she showed a good response, then a possibility of nephrectomy and radiotherapy to the mandible was to be considered later in the course.

### 2.4 Follow-up and outcomes

The patient initially responded well to sunitinib, with a significant reduction in mandibular mass size. She went on to receive a high-dose palliative radiotherapy course (30 Gy in 10#) to reduce the mandibular swelling. Unfortunately, despite the radiotherapy, her situation deteriorated and subsequent imaging showed local progression and spread to the frontal lobe of the kidney and the liver. The patient died 11 months after the initial diagnosis.

## 3 Discussion

The classic triadic presentation of RCC only occurs in 10–15% of cases and is usually indicative of advanced disease [1]. Early disease tends to be occult in nature; half the cases are now detected incidentally on radiographic examination, usually investigating an unrelated symptom [8]. The more common presentations tend to be vague and non-specific, this can cloud the clinical picture and further contribute to diagnostic delay. 25–30% of patients have metastatic spread at the time of diagnosis [9].

RCC can spread locally or via haematogenous or lymphatic systems, thus it is hard to predict where a metastatic deposit will arise. Common sites for metastasis are regional lymph nodes, lungs, bone, liver and the brain [8]. Despite metastases to the orofacial soft tissues and sinuses being well documented, involvement of the mandible is extremely rare with only a few case reports over the last 25 years [6].

This patient came to the attention of secondary care due to the persistence of her facial swelling. This was initially attributed to the more common diagnosis of parotitis and thus was being managed within the primary care setting. Whilst facial swelling is a common presentation of parotitis, it is not pathognomonic, and certainly not in isolation. A classical picture of parotitis would be recurrent painful swellings associated with meals. If an infective aetiology is suspected, one might expect to achieve exudates of pus from the parotid duct on milking. The patient could also present with pyrexia and other signs suggestive of infection or inflammation. Cranial nerve palsy is uncommon with parotitis. In this case, there was a history of trigeminal nerve involvement that suggests a more sinister cause of the facial swelling.

Following identification of the primary tumour, a focused history taking by the oncologist revealed a single episode of macroscopic haematuria 7 months prior to her facial swelling. Patients with macroscopic haematuria represent a higher-risk group than those with microscopic haematuria [10]. In fact, it is a presenting sign in over 66% of patients with urological cancer [11]. In the UK, current guidance set by the National Institute of Clinical Excellence (NICE) advises that a referral is necessary if the patient is over 45 years old and visible haematuria is either not associated with a urinary tract infection (UTI) or persists following treatment of UTI. In this case, the patients haematuria ceased following a course of antibiotic therapy.

## 4 Conclusion

Although rare, it should be kept in mind that orofacial symptoms such as mandibular swelling can be the initial presentation of systemic disease. Early diagnosis is crucial to reduce morbidity and mortality. A more sinister pathology should be considered when there is cranial nerve palsy or exclusion of an infective cause.

## Declaration of Competing Interest

None to declare.

## Funding

This research did not receive any specific grant from funding agencies in the public, commercial, or not-for-profit sectors.

## Ethical approval

Our study is exempted from ethical approval in our institution.

## Consent

Written informed consent was obtained from the patient’s next of kin for publication of this case report and accompanying images. A copy of the written consent is available for review by the Editor-in-Chief of this journal on request.

## Author contribution

Roy Zhang: writing – original draft preparation.

Chang Woo Lee: writing – review and editing, conceptualisation.

Shadi Basyuni: writing – review and editing, conceptualisation.

Vijay Santhanam: writing-review and editing, conceptualisation, supervision.

## Registration of research studies

N/A.

## Guarantor

Shadi Basyuni and Vijay Santhanam.

## Provenance and peer review

Not commissioned, externally peer-reviewed.
